# Why Reassurance Fails in Patients with Unexplained Symptoms—An Experimental Investigation of Remembered Probabilities

**DOI:** 10.1371/journal.pmed.0030269

**Published:** 2006-08-01

**Authors:** Winfried Rief, Andrea Maren Heitmüller, Katja Reisberg, Heinz Rüddel

**Affiliations:** Department of Clinical Psychology, University of Marburg, Marburg, Germany; James Cook University, Australia

## Abstract

**Background:**

Providing reassurance is one of physicians' most frequently used verbal interventions. However, medical reassurance can fail or even have negative effects. This is frequently the case in patients with medically unexplained symptoms. It is hypothesized that these patients are more likely than patients from other groups to incorrectly recall the likelihoods of medical explanations provided by doctors.

**Methods and Findings:**

Thirty-three patients with medically unexplained symptoms, 22 patients with major depression, and 30 healthy controls listened to an audiotaped medical report, as well as to two control reports. After listening to the reports, participants were asked to rate what the doctor thinks the likelihood is that the complaints are caused by a specific medical condition.

Although the doctor rejected most of the medical explanations for the symptoms in his verbal report, the patients with medically unexplained complaints remembered a higher likelihood for medical explanations for their symptoms. No differences were found between patients in the other groups, and for the control conditions. When asked to imagine that the reports were applicable to themselves, patients with multiple medical complaints reported more concerns about their health state than individuals in the other groups.

**Conclusions:**

Physicians should be aware that patients with medically unexplained symptoms recall the likelihood of medical causes for their complaints incorrectly. Therefore, physicians should verify correct understanding by using check-back questions and asking for summaries, to improve the effect of reassurance.

## Abstract 2

Those patients for whom there is no medical explanation for their symptoms are likely to have more difficulty than other patients in remembering information intended to reassure them about their condition.

## Editors' Summary

**Background.**

Being told by the doctor that that niggling headache or persistent stomach ache is not caused by a medical condition reassures most patients. But for some—those with a history of medically unexplained complaints—being told that tests have revealed no underlying cause for their symptoms provides little or no reassurance. Such patients have what is sometimes called “somatization syndrome.” In somatization, mental factors such as stress manifest themselves as physical symptoms. Patients with somatization syndrome start to report multiple medically unexplained symptoms as young adults. These symptoms, which change over time, include pain at different sites in the body and digestive, reproductive, and nervous system problems. What causes this syndrome is unknown and there is no treatment other than helping patients to control their symptoms.

**Why Was This Study Done?**

Patients with medically unexplained complaints make up a substantial and expensive part of the workload of general medical staff. Part of this expense is because patients with somatization syndrome are not reassured by their medical practitioners telling them there is no physical cause for their symptoms, which leads to requests for further tests. It is unclear why medical reassurance fails in these patients, but if this puzzle could be solved, it might help doctors to deal better with them. In this study, the researchers tested the idea that these patients do not accept medical reassurance because they incorrectly remember what their doctors have told them about the likelihood that specific medical conditions could explain their symptoms.

**What Did the Researchers Do and Find?**

The researchers recruited patients with medically unexplained symptoms and, for comparison, patients with depression and healthy individuals. All the participants were assessed for somatization syndrome and their general memory tested. They then listened to three audiotapes. In one, a doctor gave test results to a patient with abdominal pain (a medical situation). The other two tapes dealt with a social situation (the lack of an invitation to a barbecue) and a neutral situation (a car breakdown). Each tape contained ten messages, including four that addressed possible explanations for the problem. Two were unambiguous and negative—for example, “the reason for your complaints is definitely not stomach flu.” Two were ambiguous but highly unlikely—“we don't think that you have bowel cancer; this is very unlikely.” The researchers then assessed how well the participants remembered the likelihood that any given explanation was responsible for the patient's symptoms, the missing invitation, or the broken-down car. The patients with somatization syndrome overestimated the likelihood of medical causes for symptoms, particularly (and somewhat surprisingly) when the doctor's assessment had been unambiguous. By contrast, the other participants correctly remembered the doctor's estimates as low. The three study groups were similar in their recall of the likelihood estimates from the social or neutral situation. Finally, when asked to imagine that the medical situation was personally applicable, the patients with unexplained symptoms reacted more emotionally than the other study participants by reporting more concerns with their health.

**What Do These Findings Mean?**

These results support the researchers' hypothesis that people with somatization syndrome remember the chance that a given symptom has a specific medical cause incorrectly. This is not because of a general memory deficit or an inability to commit health-related facts to memory. The results also indicate that these patients react emotionally to medical situations, so they may find it hard to cope when a doctor fails to explain all their symptoms. Some of these characteristics could, of course, reflect the patients' previous experiences with medical professionals, and the experiment will need to be repeated with additional taped situations and more patients before firm recommendations can be made to help people with somatization syndrome. Nevertheless, given that medical reassurance and the presentation of negative results led to overestimates of the likelihood of medical explanations for symptoms in patients with somatization syndrome, the researchers recommend that doctors bear this bias in mind. To reduce it, they suggest, doctors could ask patients for summaries about what they have been told. This would make it possible to detect when patients have misremembered the likelihood of various medical explanations, and provide an opportunity to correct the situation.

**Additional Information.**

Please access these Web sites via the online version of this summary at http://dx.doi.org/10.1371/journal.pmed.0030269.

• MedlinePlus encyclopedia entry on somatization disorder

• Wikipedia page on somatization disorder (note that Wikipedia is a free online encyclopedia that anyone can edit)

• Prodigy Knowledge's information for patients on somatization and somatoform disorders

## Introduction

One major medical intervention by medical practitioners is the provision of reassurance that symptoms are not caused by serious medical conditions. However, in a subgroup of patients, reassurance of normal test results fails, and patients remain concerned [1]. Reassurance appears to be particularly ineffective in patients with a history of unclear somatic complaints [2]. Although the labeling of these syndromes is a point of discussion (functional syndromes, medically unexplained symptoms, somatoform symptoms, psychosomatic syndromes, etc.), we will use the term “somatization syndrome” for patients with medically unexplained symptoms in this article [3,4]. We will focus on information processing by these patients when listening and remembering medical information. Our major question is whether patients with unclear somatic complaints remember the wrong probabilities about excluded medical causes for their physical symptoms. Remembering medical reports incorrectly might not only increase the effects of health anxiety and body misperception, but may also be a source of misunderstandings between physicians and their patients.

Patients with a history of unclear somatic complaints represent a substantial subgroup of patients asking for medical treatment. Van Hemert et al. [5] found that 30% of newly admitted patients attending general medical outpatient clinics had no medical diagnosis to account for their symptoms. While single symptoms may vary, a history of multiple unclear somatic complaints typically predicts long-term persistence [6]. These patients with “somatization syndrome” are responsible for high health-care use and high treatment costs [7–9]. For the classification of somatization disorder, *International Classification of Diseases,* 10th revision, includes the criterion that medical reassurance does not have long-lasting effects. Instead these patients tend to misinterpret physical perceptions and doctors' information [10]. Barsky [11] labels this cognitive style “somatosensory amplification.”

To date, it is unclear why medical reassurance fails in this patient group. Somatic stimuli can lead to cognitive interference in these patients, with the consequence that medical information is processed and remembered less efficiently [12]. This is not caused by a deficiency in the ability to remember health-related words in general [13]. We hypothesize that patients with somatization syndrome do not have difficulties in remembering the medical terms provided by their physicians, but in remembering the likelihood of medical causes for their symptoms. To test this hypothesis, patients listened to a doctor reporting the results of a normal physical examination, and afterwards we assessed patients' memory of the doctor's thoughts of the likelihood of a serious medical condition. To assess the specificity of a false probability memory in somatization disorder, this condition is compared to two other conditions, a social rejection situation and a neutral situation (car problems). The likelihood ratings for serious interpretations in patients with somatization syndrome are compared to those of patients with major depression (which is a frequent comorbid disorder, but does not typically share the somatosensory amplification style) and to healthy controls.

## Methods

### Participants

The final sample consisted of 85 participants: 33 patients fulfilled the criteria for medically unexplained symptoms (somatization syndrome; see below), 22 patients had major depression and served as a clinical control group, and 30 participants were in the healthy control group. The groups did not differ significantly in terms of age, sex, or memory capacity. Patient recruitment took place in a hospital for mental and psychosomatic disorders in Bad Kreuznach, Germany. Healthy controls were recruited from the surroundings of the University of Marburg; healthy controls did not come from the surroundings of the hospital; the control group did not include more health professionals than the other groups.

Patients were included in the somatization group if they reported having more than seven unclear somatic complaints during the last 2 y, or if they fulfilled the criteria for hypochondriasis. Somatic complaints were assessed with the Screening for Somatoform Symptoms (SOMS) and with a structured clinical interview (see below). Clinical controls had to fulfill the criteria for major depression; they were not allowed to fulfill the criteria for somatization syndrome, even not according to the less restrictively defined Escobar criteria SSI-4/6 [14]. Healthy controls could not fulfill the criteria for somatization syndrome SSI-4/6 or any other major mental disorder.

### Assessment

For all patients, the medical records were checked and the physicians were interviewed to assess whether existing somatic complaints were medically unfounded. Patients' psychiatric diagnoses were validated using a structured clinical interview (IDCL; [15]) to confirm the diagnosis of somatization syndrome or major depression.

The SOMS [16] was additionally used to assess somatoform symptoms. This is a self-rating scale asking about 53 somatic complaints during the last 2 y (e.g., abdominal pain, nausea, palpitation, pain in extremities, back pain, dizziness, etc.). Patients were instructed to agree only if the symptom was present, doctors didn't find a sufficient explanation for the symptom, and the symptom was disabling or led to doctor visits. The 53 symptoms cover all the symptoms mentioned in ICD-10 and DSM-IV for somatization disorder and somatoform autonomic dysfunction. All positively answered symptoms are summed up to a somatization total score, which corresponds to the number of somatoform symptoms. The self-rating scale has been shown to be a good indicator for an interview-based assessment of somatoform symptoms, with correlations of *r* = 0.75 between the interview and the self-rating scale. The retest reliability of the SOMS has been found to be *r*_tt_ = 0.85 [16].

The Whiteley Index [17] is a 14-item scale asking for hypochondriacal symptoms; the answering format is binary (yes/no). The CES-D is a depression scale that is frequently used in epidemiological research [18,19]. We used the 15-item version, with answer categories between zero (rarely) and three (most of the time).

As the major experimental task depended on memory capacity, the groups were tested for memory performance using the digit span, a subtest of the Wechsler Memory Scale. It was hypothesized that patient groups would not differ in memory capacity.

### Procedure

The procedure was approved by the ethics committee of the Chamber of Medicine of the state Rheinland-Pfalz. In the hospital, patients were prescreened for somatization or depressive disorder using the SOMS and the depression scale CES-D. For those patients with increased scores, medical information was collected to check whether symptoms were medically unexplained. Patients were then given further information about the study, and informed consent was obtained from all participants. A total of 140 patients with increased depression or somatization scores were informed about the study, and 98 were willing to participate. Thirteen patients were excluded because they did not fulfill inclusion criteria for the group they were considered for.

Afterwards, patients filled in the self-rating scales and underwent the memory capacity test (digit span). Then the experimental investigation started.

### Experimental Investigation

All participants listened to three audiotapes, which were presented in a counterbalanced sequence. The tapes presented oral reports spoken by the same male voice. One report described a medical situation, one a social situation, and one a neutral situation. The duration of the three oral reports was comparable (between 2:16 and 2:28 min). All of them included concerns (about illness, about social rejection, or about damage to an expensive car). Each report included ten principle messages, including four that addressed possible explanations for the problem: two unequivocal explanations (e.g., “The reason for your complaints is definitely not a stomach flu”) and two highly unlikely, but still possible, explanations (e.g., “I don't think that you have bowel cancer; this is very unlikely”).

The medical situation was characterized by a person with abdominal pain visiting his or her doctor, who reports the results of the medical tests. The social situation was characterized by a person who learns from a friend that he or she is not invited to a neighborhood barbecue party. The neutral situation was characterized by a person having car problems, and who is informed by the mechanic about possible reasons why the motor doesn't work correctly.

After each report, participants were asked ten questions, including two (after the social situation) to four (after the medical and neutral situations) items with ratings for the remembered probabilities of reasons for the problems presented in the reports. These likelihood items were answered using a visual analog scale, ranging from zero (absolutely unlikely) to 100 (absolutely likely). Results of the likelihood items for each situation type were averaged for the individuals in each group. The remaining items were about behavioral aspects or neutral information included in the reports; they served as items distracting from the main purpose of the study, and no significant differences were found for the answers to these distracting items.

Table 1 describes some of the information presented orally by the doctor in the medical report, as well as the corresponding items asked of the participants after the presentation of the doctor's report. The table also includes examples of the other two types of reports and corresponding items.

**Table 1:**
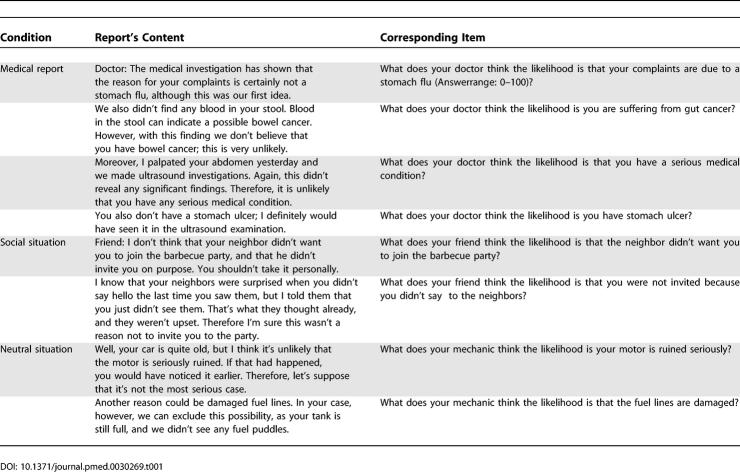
Examples of Information in Oral Reports and Corresponding Items

We hypothesized that patients with unclear somatic complaints (somatization syndrome) would remember an increased likelihood that medical conditions explain the symptoms, even though the doctor rejected most of the medical explanations in his report. The clinical and healthy controls were expected to report very low remembered probabilities. For the social situation, we expected depressive patients to be highly sensible and negative interpretations to be more likely than for the two other groups. For the neutral condition, we expected comparable ratings for all three groups.

After asking for the ratings about the likelihoods that the doctor, the neighbor, and the mechanic assign to different causes, participants also had to give cognitive and emotional appraisals, imagining themselves personally in the specific situations. The cognitive appraisals were solicited with the following questions: “How would you rate your health state after this discussion with the doctor?” (0 = absolutely healthy; 100 = absolutely sick); “What's the likelihood you would think that your neighbors didn't invite you on purpose?” (0–100); and “How seriously would you think your car is damaged?” (0–100). The emotional appraisals of all three conditions were solicited with the question: “How concerned would you be in this situation?” (0–100). It was expected that patients with somatization syndrome would be most concerned after the medical encounter, while depressives would be most concerned after the social rejection situation.

### Statistical Analysis

Data were analyzed using one-way ANOVA, with subsequent Sheffé pairwise comparisons if indicated by a significant ANOVA result. Percent data were analyzed using χ^2^ tests.

## Results

### General Aspects

The groups were comparable in terms of age, gender, and memory capacity. Therefore, any group differences for the probability ratings were not due to general memory differences. The rates for comorbid anxiety disorders were comparable between the clinical groups. As expected, the number of somatoform symptoms was significantly the highest in the somatization group, as were the scores for hypochondriasis. Patients in the somatization group were also substantially depressed, which underlines the high comorbidity between somatization and depression. Both clinical groups also showed significantly increased scores for health-care use (see Table 2).

**Table 2:**
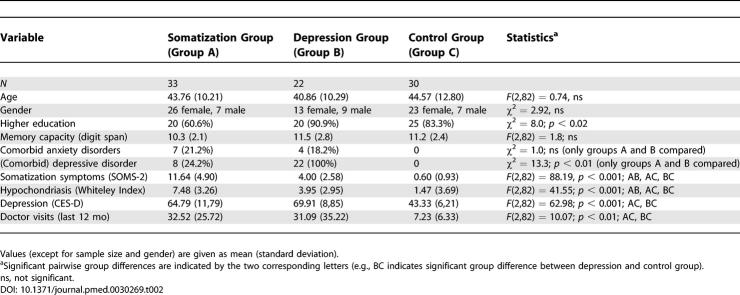
Sample Characteristics

### Likelihood Ratings

The groups differed significantly in their likelihood estimates of medical causes for the abdominal complaints, while they didn't differ in their likelihood estimates of social rejection causes in the social situation or for the neutral situation ratings. While controls remembered the doctor's probability ratings as very low, patients with unclear somatic complaints remembered the doctor's likelihood estimates of medical reasons for the complaints to be significantly higher. While we expected the depression group to show the highest scores for the social rejection interpretation of the social situation, these differences were statistically not significant, although the highest scores were found for the depression group.

We also wanted to examine whether the differences in the remembered probabilities for medical reasons were due to the doctor's statements with ambiguous versus unambiguous information. Items 1 and 4 (see Table 1) express unambiguous explanations, while items 2 and 3 express unlikely, but still possible, options. The scores in Table 3 confirm that healthy controls rated the probability of medical causes for medically unambiguous information as lower than the probability of medically ambiguous information (*t* = 2.0; d.f. 29; *p* = 0.05). This was nearly reversed for the clinical groups. For medically unambiguous information, significant differences between the groups were found, again with highest scores for the somatization group. Patients in the somatization group reported even higher probabilities for explanations unambiguously rejected by the doctor than for ambiguous explanations (*t* = 2.3, d.f. 32; *p* < 0.05); for the depression group, this comparison was not significant (*t* = 1.8; d.f. 21; not significant).

**Table 3:**
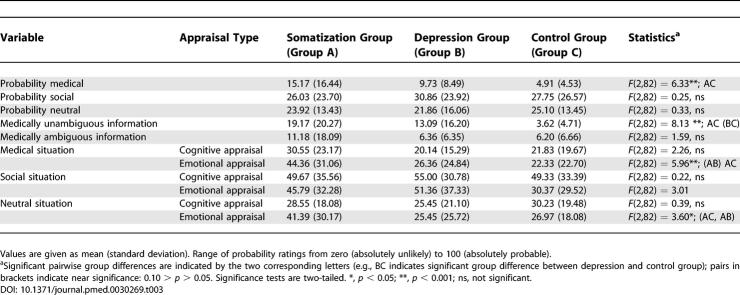
Probability Ratings

### Cognitive Appraisal

The cognitive appraisal task asked for the likelihood estimations of negative interpretations if participants were personally confronted with the presented situations. There were no significant group differences for any of the situations (see Table 3).

### Emotional Appraisal

The questions about emotional appraisal asked how concerned participants would be if they were in the situations. A highly significant effect was found for the medical situation: patients with unclear somatic complaints would find it highly concerning to suffer from abdominal pain and to get the doctor's report. However, there was also a weaker, but still significant, effect for emotional appraisal of the neutral situation: patients with somatization syndrome were also more concerned about the negative outcome of the car investigation (see Table 3).

### Further Analysis

We wanted to analyze how the overestimation of medical probabilities was related to other psychological variables. Therefore, we correlated the likelihood estimates for the medical explanations with health anxiety (Whiteley Index), depression (CES-D), and number of somatoform symptoms (SOMS). Overestimation of medical probabilities was associated with higher health anxiety (*r* = 0.26; *p* < 0.05) and with more somatoform symptoms (*r* = 0.26; *p* < 0.05), but not with depression (*r* = 0.12; not significant). This again confirms the specificity of the found effect.

## Discussion

Our results show that patients with unclear medical symptoms overestimate the likelihood of medical causes of symptoms. Although in the medical report the doctor rejected most of the mentioned medical explanations for the symptoms, the patients with unclear somatic complaints remembered higher probabilities for these medical explanations. The mean estimates of the somatization patients for the likelihood of medical explanations were about 15%, which is not very high, but still suggests that the patients had not discarded the medical causes as a possibility. Patients with medically unexplained symptoms might have a tendency to overestimate the risk of diseases in general, as was found for hypochondriasis [20,21]. This tendency might be maintained by further remembering increased likelihood estimates for medical causes. As our clinical group included only four (of 33) patients with hypochondriasis, the reported effects are not completely determined by this small subgroup, but also hold for somatization syndrome in general.

Our results are all the more striking because our items about the likelihood of explanations were not related to the patient's personal situation, but to a virtual situation. This bias in remembering likelihood estimates was only found for the medical report situation, not for the social or neutral situations. Amazingly, this effect was even more pronounced for the unequivocally rejected medical explanations, compared to the unlikely, but marginally probable, explanations.

This effect cannot be accounted for by a general memory deficit, because the groups performed equally well on the digit span test. Also a general deficit in the ability to remember illness-related information cannot explain the results. In a previous study [13], we compared 58 patients with somatization syndrome and 21 healthy controls. In a free recall memory test, participants had to report memorized words from a list including neutral and illness-related words. No group differences were found. Some contradictory results to this exist, but they found only small effects [22,23]. For somatization-associated problems, attentional biases in favor of medical information were not found [24]. Therefore, a general memory bias for illness-related information in patients with medically unexplained symptoms seems unlikely.

Further items assessed cognitive interpretation and emotional involvement if participants were confronted with the mentioned situation personally. As expected, we found highest scores for emotional involvement for the somatization syndrome group in the medical condition. Although this group also showed increased emotional appraisal scores for the neutral situation, group differences were most pronounced for the medical situation. This confirms in part the reduced capacity of patients with somatization syndrome to cope with physical misperceptions, and to cope with unexplained symptoms. It has been demonstrated that hypochondriacal and somatizing patients agree to having more thoughts about illness interpretation of bodily sensations, and that their thought content resembles catastrophic interpretations [9,10]. This threatening interpretation and subsequent attention-focusing increases the perception of pain symptoms and reduces distraction [25], leading to a vicious circle of somatosensory amplification. This vicious circle might be further amplified by the demonstrated memory bias in overestimating the probabilities of causes discussed during medical encounters.

The strength of this study seems to be the ecological validity of the study design using a typical physician explanation that test results have not revealed any pathological findings, but also the use of two comparison groups and two comparison situations. However, this study also suffers from some shortcomings. The results would be strengthened if more than one report per situation type were tested. With the procedure used in this study, the number of assessment items is limited by the number of reports and included statements. Therefore, our number of items per condition is low, and could be increased by using multiple situation presentations. Furthermore, we used medical explanations with likelihood ratings in the extreme range (“it's extremely unlikely that you have …”); although this is a frequent condition in medical practice, the psychometric properties of these extreme ratings are less optimal to show group differences. Moreover, defining somatization syndrome for patients with unclear somatic complaints is still a point of discussion [26,27]. In this study, we wanted to be on the safe side and included only patients with at least seven medically unexplained symptoms during the last 2 y. This criterion has been shown to be associated with an optimum of sensitivity and specificity in differentiating disabled patients [28,29]. As our patients were selected in a psychosomatic hospital, which is typically the ultimate step of the treatment path for this syndrome, our sample represents the highly disabled and chronically ill subgroup of patients with somatization syndrome. Further, the groups were comparable on most, but not all, demographic variables. Fewer patients in the somatization group reported higher education than patients in the other groups. However, the memory test (digit span), which is an important predictor of intellectual capacity, especially for the task used in this trial, was comparable between groups.

This study has clinical implications for the most frequently used medical intervention, namely, providing reassurance. Doctors usually expect that explaining that tests have shown no abnormality is enough to reduce health anxiety [1], but Coia and Morley [30] have summarized that medical reassurance frequently leads to harmful responses and increases in health anxiety. Our results show that medical reassurance and the presentation of negative test results can lead to patients remembering overestimated probabilities for medical explanations, especially in patients with unclear somatic complaints. Physicians should keep this effect in mind and try to reduce it. Check-back questions on what patients have understood from doctors' reports, and asking patients for summaries about the provided information, could help to detect this memory bias, and offer the opportunity to correct the remembered likelihood estimates [31]. These implications should be further addressed in future studies.

## Supporting Information

Alternative Language Abstract S1 Translation of the Abstract into German by Winfried Rief (22 KB DOC)

## Abbreviations

SOMS: Screening for Somatoform Symptoms
