# Supplementary material for: Why Reassurance Fails in Patients with Unexplained Symptoms—An Experimental Investigation of Remembered Probabilities
Source: PLoS Med. 2006 Aug 1;3(8):e269. doi: 10.1371/journal.pmed.0030269 (PMC1523375; doi:10.1371/journal.pmed.0030269)
Supplement: Supplementary file 1 [file pmed.0030269.sd001.doc]

Warum ärztliche Rückversicherung bei Personen mit medizinisch unklaren Symptomen nicht funktioniert – eine experimentelle Untersuchung erinnerter Wahrscheinlichkeiten

Hintergrund: Eine der häufigsten ärztlichen Handlungen ist die verbale Rückversicherung, dass bestimmte Beschwerden nicht durch eine schwere organische Erkrankung bedingt sind. Manchmal wirkt diese Rückversicherung auf Patienten jedoch nicht beruhigend, sondern hat ggf. sogar negative Effekte. Dies ist ganz besonders bei Patienten mit medizinisch unklaren Symptomen der Fall. Es wird die Hypothese verfolgt, dass Personen mit medizinisch unklaren Symptomen die vom Arzt präsentierte medizinische Information nicht korrekt memorieren; es wird vermutet, dass die vom Arzt geäußerte Wahrscheinlichkeit von bestimmten medizinischen Erklärungen nur verzerrt abgespeichert wird.

Methodik: 33 Patienten mit medizinisch unklaren Symptomen, 22 Patienten mit schwerer Depression und 30 Kontrollpersonen wurde über Tonband ein ärztlicher Bericht vorgespielt sowie als Kontrollbedingung zwei weitere Berichte. Nachdem die Teilnehmer diesen Berichten zugehört hatten, wurden sie gebeten, die Wahrscheinlichkeit bestimmter vom Arzt geäußerter medizinischer Erklärungen für die Beschwerden widerzugeben.

Hauptergebnisse: Obwohl in dem dargebotenen ärztlichen Bericht die meisten medizinischen Erklärungen für die körperlichen Beschwerden als unwahrscheinlich verworfen wurden, erinnern gerade Personen mit medizinisch unklaren Beschwerden eine höhere Wahrscheinlichkeit für die medizinischen Erklärungen als die Personen der depressiven und gesunden Kontrollgruppe. Dieser Gruppenunterschied findet sich hypothesenkonform nur bei den Wahrscheinlichkeitserinnerungen aus dem medizinischen Bericht, jedoch nicht bei den Kontrollbedingungen. Zusätzlich wurden die Personen über die Auswirkungen des ärztlichen Berichtes befragt, wenn es hierbei um sie selbst gegangen wäre und nicht um einen anderen Beispielpatienten. Patienten mit multiplen medizinischen Beschwerden berichten mehr Sorgen über ihren Gesundheitsstatus als die Kontrollpersonen, wenn sie sich vorstellen, dass der ärztliche Bericht ihnen gegolten hätte.

Schlussfolgerungen: Ärzte sollten sich bewusst sein, dass Patienten mit medizinisch unklaren Beschwerden die Wahrscheinlichkeitsaussagen für medizinische Erklärungen zu ihren Beschwerden falsch abspeichern. Deshalb sollte der behandelnde Arzt eine korrekte Abspeicherung der medizinischen Informationen prüfen und verifizieren, z.B. unter Verwendung von Rückfragen oder Patienten-Zusammenfassungen. Es wird erwartet, dass dadurch der beruhigende Effekt medizinischer Rückversicherung verbessert werden kann.
